# Demonstration of Japanese radiographic examination codes in establishing typical values for a wide variety of general radiography examinations

**DOI:** 10.1038/s41598-024-52294-y

**Published:** 2024-01-26

**Authors:** Ayako Yagahara, Daisuke Ando, Makoto Oda

**Affiliations:** 1 Faculty of Health Sciences, Hokkaido University of Science, Sapporo, Japan; 2 Department of Radiology, Southern TOHOKU Proton Therapy Center, Koriyama, Japan; 3 Department of Radiological Technology, Hokkaido University Hospital, Sapporo, Japan

**Keywords:** Computational biology and bioinformatics, Diagnosis, Medical imaging

## Abstract

The purpose of this study was to demonstrate Japanese radiographic examination codes JJ1017 in establishing typical values for a wide variety of general radiography. About 200,000 sets of examination data were collected, including exposure conditions, JJ1017 code applied, examination room numbers and patient information. Typical values for adults, children, and infants were calculated from the collected data, and the following items were examined: comparing typical values of general radiography in Japan DRLs 2015 and typical values in a facility; comparison of typical values between X-ray equipment for examinations of DRLs 2015; comparison of typical values for different procedures at the same anatomical site; identification of examination items associated with high radiation doses. The total numbers of JJ1017 codes applicable to the examinations were 45,372 for adults, 542 for children, and 2339 for infants. To calculate the typical values and compare these with the DRLs, we used a combination of JJ1017 anatomical codes, posture codes, and direction of radiation codes. The combination of these codes allowed the calculation of a typical value and comparison with DRLs 2015. Comparison between devices reveals differences in radiation doses and provides an opportunity to review the characteristics of the devices and their operation to suggest dose reductions. By calculating typical values for examination items for which the DRLs were not available, we were able to identify examination items with high doses in a facility and suggest items that should be audited in the facility.

## Introduction

With the increasing concerns about radiation doses in radiological examinations, activities to reduce and optimize radiation exposure are being promoted around the world. The International Commission on Radiological Protection (ICRP) has defined diagnostic reference levels (DRLs) as reference values above which some specific action or decision must be taken if a value exceeding that value is measured^1^. In Japan, DRLs2015^2^ and DRLs2020^3^ have been published by Japan Network for Research and Information on Medical Exposure (J-RIME) so far. However, as national DRL for X-ray procedures need large surveys or registries, and as these can require substantial effort to perform and analyze, they are not always responsive to changes in technology^1^. Then, where it is apparent that further optimization is being achieved locally, or where no national DRL values exist, “local DRLs or typical values” based on surveys can introduce and further assist the optimization process^1^. Local DRLs (LDRLs) set the 75th percentile value of the imaging dose at the facility has been set in consideration of differences in equipment and procedures in medical institutions in an area. The typical value is defined as the median of the distribution and used in a similar manner for smaller numbers of X-ray rooms or one facility. Typical values can be useful where a facility performs large numbers of specialized examinations for which there is no national DRL^1^.

In the implementation of LDRLs and typical values in a facility, the overall management of a wide variety of X-ray examinations will allow for efforts to reduce and optimize radiation doses. Especially, general radiography is a valuable and highly cost-effective technique for diagnosis and screening purposes in medicine to begin a procedure, constituting the largest contribution worldwide^4^ and it is necessary to manage and control optimal exposure doses. Previous studies on LDRLs and typical values have been investigated for pelvic examinations for adults^5^, mammography^6,7^, adult and pediatric chest X-rays^8,9^, and these studies have focused on specific examinations. There are also studies that addressed six different test items for the trunk^10^ and items focused on in the Japanese DRLs 2015^11^, but these studies examined major or high-dose examinations and did not address a wide variety of procedures.

To collectively manage a variety of examinations, introducing an imaging procedure code is helpful. Codes are compact labels used by computer systems to identify unique concepts or codes of information and it is efficient to manage the LDRLs and typical values collectively. With regard to imaging studies, there are two types: one is procedure codes, which are identifiers for the type of examination, and the other is access numbers, which are identifiers for the specific instance of an examination^12^. In this study, we focused on the former code designations, because JJ1017 is introduced as the standard radiological examination code in Japan. This code is a structured 32-bit code that is used in combination with the codes for modality, anatomical site, procedure, and projection. This code is also intended for use in dose management. Studies using this code have been conducted using code extensions^13^ and introducing these into the radiation therapy information system and in support for radiation therapy prescriptions^14^. However, there are few papers on the application of JJ1017 to radiation exposure control. With DRLs and Local DRLs the usage of JJ1017 has not yet become standardized, and calculating LDRLs for a wide range of procedures is a challenging task for general radiography, where there are variations in procedures from facility to facility. To improve on this, the purpose of this study is to demonstrate the calculation of typical values in the wide variety of examinations taking place and evaluate the applicability of JJ1017 in one facility.

## Materials and methods

### JJ1017 code

The JJ1017 code^15^ became the only standard master for radiological regions in all of Japan in 2012 by the Ministry of Health, Labour and Welfare (MHLW) that has been recognized as an industry standard criterion in the field of health and medical informatics^16^. This is the code used for radiation appointments, accounting, and records in routine medical care and its code facilitates integration with various medical systems and medical imaging modalities.

**Figure 1:**
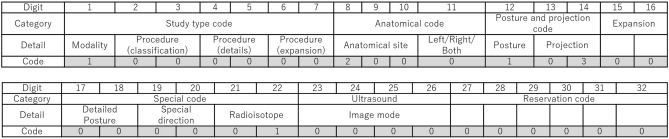
Structure of the JJ1017 code. The numbers at the bottom of the table represent codes for the chest posteroanterior (PA) view in the standing position.

Figure 1 shows the structure of JJ1017. The first 16 bits contain information of study type, anatomical site, posture, and projection. The second 16 bits contain special codes, including details of posture, special direction and radioisotope, and codes for ultrasound. The following is a detailed description of the code. The study type code is a combination of a modality code and procedure codes. Modality code “1” is conventional radiography. The procedure code is divided into classification, details, and extensions. For classification, it represents broad categories and derivatives of examinations and treatments for the same type of modality, such as “stereo radiography” or “panoramic radiography”. The details of a code can be used to elaborate on the examination and treatment techniques. The extension code is assigned as a unique code to the facility as necessary.

Anatomical code consists of the anatomical site and left and right code, and the anatomical site is represented by a three-digit code. The code for left and right is assigned like “B” for both sides, “R” for the right side, and “L” for the left side. Posture and projection code consists of posture and projection codes, and the posture code is assigned as standing, supine, or prone, and others. The projection code includes anteroposterior (AP) and posteroanterior (PA), and special imaging methods such as “Lauenstein view”.

For the latter 16 digits of the complete code, the Special code is a combination of the detailed posture, special direction, and radioisotope. The detailed postures include items focusing on joint movements, such as for intermediate, external, and internal postures. The special instruction includes codes such as stereoscopic imaging, and weighted-bearing views. The radioisotope code includes the X-ray used in diagnostic imaging and radiation therapy, the electron beam used in radiation therapy, and the radioisotopes used in nuclear medicine. The ultrasound code includes imaging modes, such as pulse doppler, and procedures, such as elastography. Reservation codes are codes that the JJ1017 committee is preparing for future expansions. In this study, Version 3.3, which was installed at the facility involved here, was used.

### Data collection

A total of 197 thousand X-ray examination records were collected at Hokkaido University Hospital from October 2013 to March 2015, and we extracted exposure conditions, tube voltages, tube currents, exposure times, JJ1017 codes, room number of the examination, and patient age as recorded in the radiological information system (RIS). The list of the X-ray tubes and image receivers utilized in each imaging room are delineated in Table 1.

**Table 1:** X-ray equipment in the examination rooms.

Room No	X-ray tube	Detector (standing position)	Detector (supine position)
1	HITACHI Radnext	FUJI CALNEO U	FUJI CALNEO 1417 wireless SQ
2	HITACHI Radnext	FUJI CALNEO U	FUJI CALNEO 1417 wireless SQ
3	SHIMADZU RAD speed pro	FUJI CALNEO mini Wireless SQ	–
4	SHIMADZU RAD speed pro	–	FUJI CALNEO 1417 wireless SQ
5	SHIMADZU RAD speed pro	FUJI CALNEO mini wireless SQ	FUJI CALNEO mini wireless SQ
6	SHIMADZU 0.6/1.2P324DK-125	FUJI BENEO	FUJI BENEO
7	SHIMADZU RAD speed Pro	FUJI CALNEO MT	FUJI CALNEO 1417 wireless SQ
8	Hitachi medical corporation U-6GE-410 TB	FUJI CALNEO 1417 wireless SQ	FUJI CALNEO 1717 wireless

### Calculation of entrance surface doses

An entrance surface dose (ESD) was estimated for each specific examination by the numerical dose determination (NDD) method using the recorded exposure conditions. The numerical dose determination (NDD) method^17^ calculates the ESD using the parameters of the tube voltage, tube current—exposure time product, distance from the focus to the skin, and the total filtration and back scatter. Coefficients for the NDD are obtained from the tube voltage and Al filtration of the X-ray spectrum. This method is accepted in Japan as a tentative substitute for dosimeter values in the absence of dosimeters^2,3^. The dose is estimated by the following equation.

$${\text{ESD}}\left( {{\text{mGy}}} \right) = {\text{NDD}} - {\text{M}}\left( {\text{f}} \right) \times {\text{mAs}} \times \left( {{1} - {\text{FSD}}} \right)^{{2}}$$

Where, NDD- M(f) is a constant determined by the total filtration and tube voltage, mAs is the product of the tube current and time, and FSD is the exposure distance^17^. In this study, the value of NDD M(f) was determined from a conversion table based on the tube voltage and filter thickness in ref^17^. Filters were aluminum for all instruments; FSD was two meters for chest and abdominal examinations in the standing position in adults and children, 1.5 m for chest and abdominal examinations in the supine position for adults and children, 1 m for infant examinations, and 1.2 m for all others.

### Calculation and evaluation of typical values

In this study, the following were demonstrated for dose control by typical values using JJ1017.

1. Calculation of typical values and comparisons between typical values and national DRLs which are publicly available
2. Calculation and comparison of typical values for the different examination rooms, which refer the differences in equipment, for the same examination items
3. Calculation of typical values for examinations without DRLs

In (1), the examination items corresponding to DRLs2015 were extracted using JJ1017. The items in DRLs consist of the anatomical site and direction of the X-ray beam, such as “Chest PA (posteroanterior)”. In order to extract examination data which corresponded to items of the DRLs, “anatomical code,” “posture code” and “projection code” in JJ1017 were used. In the estimates of typical values for adults, the dose data were collected from patients weighing 50 to 60 kg, which is the typical body size for Japanese^2^. For children and infants 5-year-olds and 12-months and younger, all collected data available were used. This study included examination items that were performed for more than 20 cases, because 20–30 cases or more, at least, were needed to calculate typical values^1^. The typical values were determined by calculating the median values of ESD for each item, based on the codes collected under the aforementioned conditions. Considering the period of data collection, the typical values were compared to the DRLs 2015 in Japan. In (2), the typical values calculated in (1) were linked to examination room numbers extracted from the RIS, and we performed the Steel–Dwass test for three groups, and the Wilcoxon rank-sum test was conducted for two groups using JMP Pro11. In (3), examination items, which were preformed 20 or more times and were not listed in DRLs 2015, were extracted and typical values were calculated.

### Ethics approval

The approval of the Ethics Committee of Hokkaido University Hospital and the Ethics Committee of Hokkaido University of Science has been obtained. This research was performed in accordance with relevant guidelines and regulations.

### Consent to participate

Informed consent was obtained in the form of opt-out on the Hokkaido University Hospital website. Those who rejected participation were excluded.

## Results

### Comparison between typical values and national DRLs

The total numbers of JJ1017 codes applicable to the examination items for which there were DRLs were 45,372 for adults, 542 for children, and 2339 for infants. There were 15 examination items which were performed 20 or more times. The JJ1017 codes and typical values for these 15 items are shown in Table 2. Relative to the DRLs2015, the estimated ESDs for each examination exhibited a range from 15 to 57% of the values in the DRLs. There were three examination items that did not reach 20 cases (forearms and two obstetric examination, Martius and Guthman), and for these typical values were not calculated.

**Table 2:** Details of typical values and DRLs 2015.

Examination items	The number of examinations	Anatomical site (code)	Posture (code)	Projection (code)	Typical value (mGy)	DRLs 2015 (mGy)
Skull	151	Cranial bones (110)	Unspecified (0)	Frontal-AP (02)/Frontal -PA (03)	0.574	3
Skull LAT	146	Cranial bones (110)	Unspecified (0)	RL(05)/ LR(06)	0.313	2
Cervical spine	353	Cervical spine (351)	Unspecified (0)	Frontal-AP (02)	0.405	0.9
Thoracic spine	99	Thoracic spine (353)	Unspecified (0)	Frontal-AP (02)	1.062	3
Thoracic spine LAT	127	Thoracic spine (353)	Unspecified (0)	Lateral (04)	1.350	6
Chest PA	13352	CHEST (200)	Standing (1)	Frontal-PA (03)	0.172	0.3
Abdomen AP supine	2594	ABDOMEN (250)/KUB(251)	Supine (2)	Frontal-AP (02)	0593	3
Infant hip joint (0-1y)	330	Hip joint (405)	Supine (2)	Frontal-AP (02)	0.063	0.2
Infant chest (0–1 y)	1006	CHEST (200)	Standing (1)/Supine (2)	Frontal-AP (02)/Frontal-PA (03)	0.063	0.2
Child chest (5 y)	196	CHEST (200)	Standing (1)/Supine (2)	Frontal-AP (02)/Frontal-PA (03)	0.102	0.7
Lumbar spine	665	Lumbar spine (354)	Unspecified (0)	Frontal-unspecified (01)	1.392	4
Lumbar spine LAT	1196	Lumbar spine (354)	Unspecified (0)	Lateral (04)	2.583	11
Pelvis	38	PELVIS (320)	Unspecified (0)	Frontal-AP (02)	0.834	3
Femur	89	Femur (407)	Unspecified (0)	Frontal-unspecified (01)	0.493	2.0
Ankle joint	131	Ankle joint (413)	Unspecified (0)	Frontal-unspecified (01)	0.084	0.2

PA, posteroanterior; AP, anteroposterior; LAT, lateral; KUB, kidney, ureter and bladder.

### Typical values for different examination rooms

**Figure 2:**
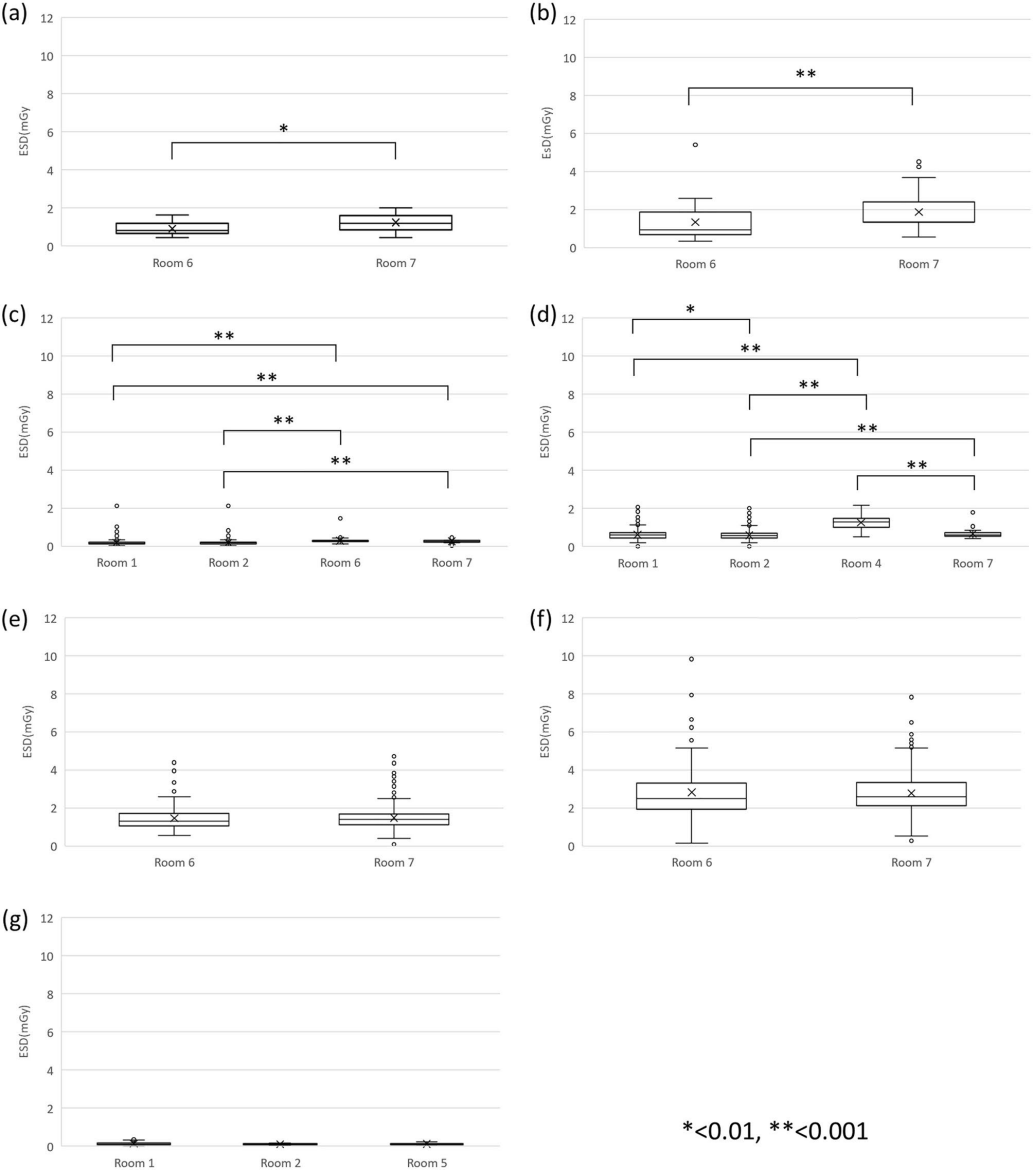
Boxplot of ESD in six procedures in X-ray examinations in specific examination rooms. (**a**) Thoracic spine, (**b**) Thoracic spine LAT, (**c**) Chest PA, (**d**) Abdomen AP supine, (**e**) Lumbar spine, (**f**) Lumbar spine LAT, (**g**) Child chest.

Among the examinations, there were seven examinations with more than 20 imaging cases in two or more examination rooms. Typical values for each examination room are shown in Table 3. Figure 2 shows the distribution of ESD in the imaging rooms represented as boxplots. No significant difference was found in the lumber supine, lumbar spine LAT and child chest.

**Table 3:** Typical value for seven procedures in different examination rooms (mGy).

	Room 1	Room 2	Room 3	Room 4	Room 5	Room 6	Room 7	Room 8
Thoracic spine	–	–	–	–	–	0.822	1.208	–
Thoracic spine LAT	–	–	–	–	–	0.937	1.349	–
Chest PA	0.159	0.172	–	–	–	0.265	0.247	–
Abdomen AP supine	0.593	0.564	–	1.279	–	–	0.610	–
Lumbar spine	–	–	–	–	–	1.311	1.392	–
Lumbar spine LAT	–	–	–	–	–	2.503	2.602	–
Child chest	0.110	0.102	–	–	0.097	–	–	–

– No or less than 20 cases in the relevant examination room.

PA, posteroanterior; AP, anteroposterior; LAT, lateral.

### Typical values for examinations for which national DRLs have not been set

The number of codes for which no DRLs was set was 164. Of these, 85 tests were performed on adults, 8 on children, and 15 on infants all in 20 or more cases. Tables 4, 5 and 6 show the top 10 tests with higher typical values in adults and infants, and all tests in children. In adults, typical values in the paranasal sinus and lateral and oblique imaging of the body trunk appeared at the top of the list. In particular, the item “thoracolumbar junction LAT” had the highest typical value among all the examinations at the facility. In children, typical values in the body trunk, including the whole spine, chest, and abdomen, were higher. Typical values in infants were also higher in body trunk including chest, abdomen, and hip joints.

**Table 4:** Typical values for examinations in adults for which National DRLs have not been set.

Examination items	Anatomical site (code)	Posture (code)	Projection (code)	Typical value (mGy)	Number of examinations
Thoracolumbar junction LAT	Thoracolumbar junction (674)	Unspecified (0)	Lateral (04)	1.808	216
Abdomen RL standing	Abdomen (250)	Standing position (1)	Lateral-RL (05)	0.829	21
Lumber spine LPO	Lumbar spine (354)	Unspecified (0)	LPO (12)	1.505	130
Lumber spine RPO	Lumbar spine (354)	Unspecified (0)	RPO (13)	1.352	128
Rib oblique view	Ribs (379)	Unspecified (0)	Oblique (09)	2.510	38
Waters’ view in paranasal sinus	Paranasal sinus (116)	Unspecified (0)	Waters (47)	1.127	85
Abdomen PA standing	Abdomen (250)	Standing position (1)	Frontal-PA (03)	0.558	4162
Rib frontal view	Rib (379)	Unspecified (0)	Frontal-unspecified (01)	1.829	46
Abdomen (KUB) PA standing	KUB (251)	Standing position (1)	Frontal-AP (02)	0.558	84
Thoracolumbar junction AP	Thoracolumbar junction (674)	Unspecified (0)	Frontal-unspecified (01)	0.923	223

**Table 5:** Typical values of examinations in children for which National DRLs have not been set.

Examination items	Anatomical site (code)	Posture (code)	Projection (code)	Typical value (mGy)	Number of examinations
Whole spine PA standing	Whole spine (678)	Standing position (1)	Frontal-PA (03)	0.089	23
Abdomen PA standing	Abdomen (250)	Standing position (1)	Frontal-PA (03)	0.153	27
Chest RL standing	Chest (200)	Standing position (1)	Lateral-RL (05)	0.144	25
Hip joint AP	Hip joint (405)	Unspecified (0)	Frontal-AP (02)	0.177	20
Hand frontal view	Hand (391)	Unspecified (0)	Frontal-unspecified (01)	0.024	21
Finger	Finger (689)	Unspecified (0)	Frontal and lateral (A3)^†^	0.018	50

^†^Frontal and lateral (A3): Facility unique code.

**Table 6:** Typical values of examinations in infants for which National DRLs have not been set.

Examination items	Anatomical site (code)	Posture (code)	Projection (code)	Typical value (mGy)	Number of examinations
Abdomen AP supine	Abdomen (250)	Supine position (2)	Frontal-AP (02)	0.125	37
Chest RL standing	Chest (200)	Standing position (1)	Lateral-RL (05)	0.112	88
Hip joint LAT	Hip joint (405)	Unspecified (0)	Lateral (04)	0.083	400
Clavicle LAT	Clavicle (377)	Unspecified (0)	Lateral (04)	0.056	36
Clavicle frontal	Clavicle (377)	Unspecified (0)	Frontal-unspecified (01)	0.053	54
Tarsus frontal view	Tarsus (706)	Unspecified (0)	Frontal-unspecified (01)	0.028	20
Hand frontal view	Hand (391)	Unspecified (0)	Frontal-unspecified (01)	0.021	50
Finger	Finger (689)	Unspecified (0)	Frontal and lateral (A3)^†^	0.019	22
Femur frontal view	Femur (407)	Unspecified (0)	Frontal-unspecified (01)	0.052	29
Tarsus frontal view	Tarsus (706)	Unspecified (0)	Lateral (04)	0.024	26

^†^Frontal and lateral (A3): Facility unique code.

## Discussion

### Use of JJ1017 codes in the calculation of typical values

The JJ1017 is a standard code approved by the MHLW, and consistency of items is ensured in the long-term storage and dose information management. In addition, when this code will be introduced more widely in other facilities, it will save time in the data curation, and will facilitate real-time detection of excess radiation exposure cases, comparison among facilities, and setting of typical values. Especially considering that general X-ray imaging involves more diverse and complex techniques compared to other radiographic examinations, we believe that setting typical values based on the JJ1017 code is useful.

As shown in Table 2, there were cases where a single concept could be assigned multiple codes. For example, the DRL for “abdomen” corresponds to “abdomen (250)” and “KUB (251)” in JJ1017. At the facility, 'abdomen' means that the upper border of the imaging range ensures the depiction of the diaphragm for the evaluation of upper abdominal organs such as the liver, and 'KUB' means that the lower border of the imaging range guarantees the depiction of the pubic bone for the evaluation of the range from the kidneys to the bladder. In addition, it is necessary to check beforehand whether there are multiple JJ1017 codes corresponding to the examination items in DRLs because the representations in JJ1017 codes are more detailed.

This study focused on only X-ray examinations, but JJ1017 also contains codes for other types of radiological examinations, such as computed tomography and angiography, and specific procedure codes corresponding to each modality are also included. Therefore, JJ1017 can be used for detailed dose control in any modality. In addition, mapping of JJ1017 to the LOINC/RSNA Radiology Playbook^18^, which is used in the dose index registry organized by the American College of Radiology, will allow international comparisons of DRLs and other representative values. With these international comparisons of DRLs and other representative values may become possible. When setting typical values, it is necessary to collect patient information such as age and weight, which are not included in JJ1017, and the examination room number for comparisons of X-ray equipment. Therefore, systems that can centrally manage such information together with the radiology department system is needed.

### Utilization of typical values for reducing radiation exposure in medical settings

By calculating typical values for each examination, it is possible to understand the radiation dose at the facility and consider the need for periodic review and corrective measures of the exposure conditions. Previous studies have reported that DRLs have shown a decrease in values over time^19,20^. Therefore, periodic re-calculation of typical values and comparison with the DRLs is expected to contribute to dose reductions in a facility.

Typical values were calculated for each examination room for the same examination items, and multiple comparisons were conducted to evaluate whether there were significant differences in the ESD between the rooms. As a result, there were examinations where significant differences were observed (Fig. 2). This observed discrepancy could be attributed to the lower output of the X-ray tube compared to other examination rooms, which may have led the radiological technologists to perceive a relative decline in image quality. Consequently, this perception possibly prompted an extension of the imaging duration and the establishment of exposure conditions that inadvertently increased the radiation dose. It was also considered that the type and use of auxiliary equipment such as grids for scatter X-ray removal, which are necessary for obtaining high-quality X-ray images, may affect the increase in radiation doses. Statistical analysis of ESD in multiple examination rooms and comparisons of typical values can provide an opportunity to reflect on the characteristics and operational particulars of the equipment, which can also contribute to the audit cycle.

The European Society of Radiology recommends setting LDRLs even if national DRLs are not publicly available^21^. Therefore, typical values for examination items without established DRLs in the facility were calculated in this study. We found that some examination items had a higher typical value than those with established DRLs. This result indicates that calculating the typical value for each examination is important even if DRLs are not publicly available. Additionally, it was found that the examinations with a consistently high typical value among adults, children, and infants were those that involve lateral views of the body trunk. This is likely due to the thicker body thickness and longer X-ray transmission distances leading to higher doses. Previous studies^5,10^ have also mentioned the importance of dose management in the body trunk area, as the reproductive systems may also be included in the exposure field, and it is necessary to focus on both the frontal view as well as on the high-dose lateral view.

## Limitations

Limitations of this study were as follows: NDD may potentially underestimate the dose due to a lack of comprehensive evaluation of the irradiation field size. Data collection was conducted for a limited period and typical values in this study were not comparable to the most recent Japan DRLs. The weight data may not have been updated in real time for outpatients. The study was conducted at a single facility.

## Conclusions

This study demonstrated the usefulness of the JJ1017 codes in establishing typical values for a wide range of general X-ray examinations, and the following items were examined: comparison of typical values of general radiography between Japan DRLs 2015 and typical values in a facility; comparison of typical values between X-ray equipment for examination items of DRLs 2015; identification of examinations associated with high typical values. Combination of anatomical codes, posture codes, and direction of radiation codes allowed the calculation of typical values and comparisons with DRLs 2015. The comparisons of devices show differences in irradiation doses and provides an opportunity to review the characteristics of the devices and their operation for dose reductions. By calculating typical values for examination items for which DRLs were not available, we were able to identify examination items with high doses in facilities and identify items that should be audited in facility.

## Data Availability

The data that support the findings of this study are available from Hokkaido University Hospital, but restrictions apply to the availability of these data, which were used under license for the current study, and so are not publicly available. Data are however available from the corresponding authors upon reasonable request and with permission of Hokkaido University Hospital.
